# D-galactan II is an immunodominant antigen in O1 lipopolysaccharide and affects virulence in *Klebsiella pneumoniae*: implication in vaccine design

**DOI:** 10.3389/fmicb.2014.00608

**Published:** 2014-11-19

**Authors:** Pei-Fang Hsieh, Meng-Chuan Wu, Feng-Ling Yang, Chun-Tang Chen, Tzu-Chi Lou, Yi-Yin Chen, Shih-Hsiung Wu, Jin-Chuan Sheu, Jin-Town Wang

**Affiliations:** ^1^Department of Microbiology, National Taiwan University College of MedicineTaipei, Taiwan; ^2^Department of Internal Medicine, National Taiwan University HospitalTaipei, Taiwan; ^3^The Institute of Biological Chemistry, Academia SinicaTaipei, Taiwan

**Keywords:** D-galactan II, *Klebsiella pneumoniae*, lipopolysaccharide, immunodominant antigen, vaccine

## Abstract

In the O1 strain of *Klebsiella*, the lipopolysaccharide (LPS) O-antigen is composed of D-galactan I and D-galactan II. Although the composition of the O1 antigen of *Klebsiella* was resolved more than two decades, the genetic locus involved in the biosynthesis of D-galactan II and the role of D-galactan II in bacterial pathogenesis remain unclear. Here, we report the identification of the D-galactan II-synthesizing genes by screening a transposon mutant library of an acapsulated *Klebsiella pneumoniae* O1 strain with bacteriophage. *K. pneumoniae* strain deleted for *wbbY* exhibited abrogated D-galactan II production; altered serum resistance and attenuation of virulence. Serologic analysis of *K. pneumoniae* clinical isolates demonstrated that D-galactan II was more prevalent in community-acquired pyogenic liver abscess (PLA)—causing strains than in non-tissue-invasive strains. WbbY homologs, WbbZ homologs, and lipopolysaccharide structures based on D-galactan II also were present in several Gram-negative bacteria. Immunization of mice with the *magA*-mutant (K^−^_1_ O_1_) (that is, with a LPS D-galactan II-producing strain) provided protection against infection with an O1:K2 PLA strain. Our findings indicate that both WbbY and WbbZ homologs are sufficient for the synthesis of D-galactan II. D-galactan II represents an immunodominant antigen; is conserved among multiple species of Gram-negative bacteria and could be a useful vaccine candidate.

## Introduction

Several Gram-negative bacterial pathogens decorate their surfaces with exopolysaccharide (EPS), including capsular polysaccharide (CPS) and lipopolysaccharide (LPS). LPS is composed of lipid A, core oligosaccharide, and O antigenic polysaccharide. The O antigen contributes to serum resistance of several Gram-negative bacteria and also is a determinant of bacterial virulence (Krishnapillai, 1971; Taylor, 1976; Schneider et al., 1982; Tomas et al., 1986; Hong and Payne, 1997; Hsieh et al., 2012).

*K. pneumoniae* is an opportunistic pathogen that causes several kinds of infections, including pneumonia, bacteremia, urinary tract infection, and community-acquired pyogenic liver abscess (PLA) (Podschun and Ullmann, 1998; Ko et al., 2002; Ramphal and Ambrose, 2006; Tsai et al., 2008). There are 9 O antigens found in *Klebsiella* species, of which O1 is the most common serotype among clinical *K. pneumoniae* isolates (Alberti et al., 1993; Hansen et al., 1999). Our previous study showed that among *K. pneumoniae* causing PLA, the O1 serotype is highly predominant, with a prevalence rate of 90% (Hsieh et al., 2012). The O1 antigen contains two domains: D-Gal I and D-Gal II. The low-molecular-weight (LMW) D-Gal I polymers, which are directly linked to the core oligosaccharide, are composed of repeat units of the structure →3)-β-D-Gal*f*-(1→3)-α-D-Gal*p*-(1→. The *wb* (*rfb*) cluster, which is composed of six genes (*wzm, wzt, glf, wbbM, wbbN*, and *wbbO*), is required for the production of D-Gal I (Whitfield et al., 1991; Clarke and Whitfield, 1992). The high-molecular-weight (HMW) D-Gal II polymers, which are linked to the distal ends of D-Gal I polymers, consist of repeat units of the structure →3)-α-D-Gal*p*-(1→3)- β-D-Gal*p*-(1→, and are required for the resistance of *K. pneumoniae* to complement-mediated serum killing (McCallum et al., 1989). However, the location and identity of the genes required for D-Gal II biosynthesis are still unknown.

Bacteriophages target different capsule- or O- types of *K. pneumoniae* by recognizing surface molecules. *Klebsiella* bacteriophage O1-A, which infects the non-capsulated *K. pneumoniae* KD2 strain, has been used to screen and identify LPS mutants with spontaneous chromosomal mutations (McCallum et al., 1989). O1-A phage-resistant KD2 mutants have been classified into 2 basic types: those lacking high-molecular-weight (HMW) LPS and those lacking both LMW- and HMW-LPS. However, phage screening did not identify the genetic determinants of D-Gal II biosynthesis in the *K. pneumoniae* O1 strain (McCallum et al., 1989).

In the present study, we performed a bacteriophage screen of LPS-deficient mutants of an acapsulated *K. pneumoniae* O1 strain; these mutants had been derived from the parent strain by transposon mutagenesis. Using this screen, we identified and characterized the genetic locus involved in the biosynthesis of D-Gal II of the O1 antigen; examined the distribution of the D-Gal II antigen in PLA and non-tissue-invasive *K. pneumoniae* clinical isolates; investigated the distribution of the D-Gal II-synthesizing genes in *E. coli* O19 and other Gram-negative bacteria; explored the role of D-Gal II of *K. pneumoniae* in serum-killing and the pathogenesis of bacteremia; and investigated whether antiserum raised against LPS D-Gal II protected against D-Gal II-producing bacterial infection.

## Materials and methods

### Ethics statement

The study was approved by the Research Ethics Committee (REC) of the National Taiwan University Hospital (NTUH) (IRB approval #: 9561701018). All animal procedures were approved under application number 20140062 of the Institutional Animal Care and Use Committee (IACUC) of the National Taiwan University College of Medicine (NTUCM). Procedures were consistent with the recommendations of the *Guide for the Care and Use of Laboratory Animals* of the National Institutes of Health and Taiwan's Animal Protection Act.

### Bacterial strains and culture conditions

The bacterial strains used in this study are listed in Table S1. The *K. pneumoniae* NTUH-K2044 was isolated from a patient with liver abscess-complicated meningitis and endophthalmitis (Fang et al., 2004). The K-antigen *Klebsiella* reference strains were obtained from the Statens Serum Institute (Copenhagen, Denmark). A total of 74 clinical isolates of *K. pneumoniae* were collected from 1997 to 2003 in the National Taiwan University Hospital, as described previously (Hsieh et al., 2008). *K. pneumoniae* and *E. coli* strains were cultured in Luria-Bertani (LB) medium or LB medium supplemented with appropriate antibiotics, including 100 μg/mL ampicillin, 50 μg/mL kanamycin, or 100 μg/mL chloramphenicol.

### Isolation of *K. pneumoniae* bacteriophage O1-1

The *magA* deletion mutant was co-incubated with water from rivers of Taiwan in LB broth overnight. After centrifugation, the supernatant was filtered using a 0.45-μm filter and spotted onto LB plates overlaid with the *magA* mutant to detect phage plaques. An agar overlay method was used for isolation of a pure phage preparation and to determine phage titers as described elsewhere (Pieroni et al., 1994).

### Construction of mutant library of the *magA* mutant

A mutant library of the *magA*-deletion strain was constructed using random mutagenesis as previously described (Fang et al., 2004).

### Screening for phage-resistant mutants from the *magA*-deletion transposon mutant library

Lytic activity of O1-1 bacteriophage against the *magA*-deletion transposon mutants was measured with a spot test. A volume of 1 μL of 10^3^ plaque forming units (PFU) of the O1-1 bacteriophage was used; the sensitivity of the *magA*-deletion transposon mutants against the O1-1 was observed by formation of a clear circular zone.

### Semi-random PCR and DNA sequencing

Transposon-insertion sites were determined by semi-random PCR and DNA sequencing as described elsewhere (Chun et al., 1997). The primer pairs for semi-random PCR are listed in Table S1.

### Determination of the transcription initiation sites of *wbbY* and *wbbZ* genes

5′-Rapid Amplification of cDNA Ends (RACE) -PCR was performed using the SMARTer RACE cDNA Amplication Kit (Clontech Laboratories) following the manufacturer's instructions.

### Gene deletion and complementation

*K. pneumoniae* mutated in *wbbY, wbbZ* [polar and non-polar (NP)], *magA wbbY* (double mutant), or *wza wzb* (double mutant) were constructed using the previously described unmarked deletion method (Hsieh et al., 2008), which employs electroporation and selection with a temperature-sensitive vector (pKO3-Km) containing flanking regions for each target gene (Simon et al., 1983). For *trans*-complementation, *wbbY, wbbZ* with the intergenic region, *wbbY* with its intact promoter, or the entire *wbbY-wbbZ* region from *K. pneumoniae* NTUH-K2044 and *E. coli* F8188-41 were amplified by PCR and cloned into the pACYC184 vector (Fermentas). The recombinant pACYC184-derived plasmids were transformed into the *wbbY, wbbZ, wbbZ-NP*, and *wbbY magA* (double-mutant) strains by electroporation. The primer pairs for the deletion and complementation constructs are listed in Table S1. All sequence data for the *wbbY-wbbZ* regions from NTUH-K2044 and F8188-41 have been deposited (GenBank accession numbers KJ451390 and KJ451391).

### Bacterial growth assays

An 18-h culture of each strain was used to inoculate each 5-ml LB broth aliquot at a ratio of 1:100. Each culture was grown at 37°C and growth was monitored hourly by serial dilution and plating to LB agar with next-day quantitation of colony-forming units (CFU).

### Extraction and quantification of capsular polysaccharides (CPS)

The amount of K1 CPS from each strain was determined by assaying uronic acid content as described by Domenico (Domenico et al., 1989).

### LPS analysis

The exopolysaccharide (EPS) extracts (containing both CPS and LPS) were purified by a modified hot water–phenol extraction method, as described previously (Chuang et al., 2006; Hsieh et al., 2012). Samples were separated by 14% sodium dodecyl sulfate polyacrylamide gel electrophoresis (SDS-PAGE) containing 4 M urea (Tsai and Frasch, 1982) and visualized by silver staining (Tsai and Frasch, 1982). For immunoblots, samples were separated by SDS-PAGE and blotted to a Hybond-C membrane (Amersham, Little Chalfont, UK). Anti-D-Gal II antiserum was obtained from a rabbit immunized with a *magA*-mutant *K. pneumoniae* as described elsewhere (LTK BioLaboratories, TW); this serum was diluted 1/50,000 for LPS D-Gal II analysis.

### NMR spectroscopy

Proton spectra were carried out at 500 MHz at 25°C on solutions formulated in D_2_O by using a spectral width of 8.5 KHz, a 32 K data block, and a 90° pulse in a Bruker AVII-500 LC-NMR (Billerica, Massachusetts, USA) spectrometer with standard pulsed sequences and Bruker software.

### *wbbY* and *wbbZ* detection

In order to detect *wbbY* and *wbbZ* homologs in various species, PCR was performed using primer pairs specific for *wbbY* and *wbbZ*. Primers used in this study are listed in Table S1. PCR was performed as described elsewhere (Chuang et al., 2006).

### Serum killing assays

The survival of exponential-phase bacteria in non-immune human serum was measured as previously described (Hsieh et al., 2012). Briefly, a log-phase inoculum of 2.5 × 10^4^ CFU was mixed at a 1:3 or 3:1 vol/vol ratio with mixed non-immune human serum donated by 5 healthy volunteers. The final mixture, comprising 75 or 25% non-immune serum by volume, was incubated at 37°C for 3 h. For time-course studies, the bacteria/serum mixture, comprising 75 or 25% non-immune serum by volume, was incubated at 37°C for 1, 2, and 3 h. The colony count was determined by plating of serial dilutions on LB agar, and the mean survival ratio was plotted. A mean survival ratio ≥1 corresponds to serum resistance.

### Mouse inoculation experiments

Virulence was evaluated by mortality in a murine model of septicemia generated by intraperitoneal (IP) injection. Groups of five-week-old female BALB/c mice were infected IP with the *K. pneumoniae* NTUH-K2044 (or the isogenic mutants) in 0.1 mL of 0.95% saline (1 × 10^2^ CFU/mouse; 8 mice for each group). The exact inoculation dose was confirmed by serial dilution and plating to LB agar and survival rate of mice was monitored for 4 weeks. For *in vivo* competition, the *wbbY* mutant strain and the fully virulent p*lacZ* deletion mutant were grown (separately) to log-phase in LB broth, mixed at a 1:1 ratio (the initial dose was 1 × 10^3^ CFU each) in 100 μL sterile saline, and inoculated IP into 5-week-old BALB/c mice (8 for each group). The mice were sacrificed at 24 h post-inoculation, and the spleen and liver were removed and homogenized in 1x PBS. The concentrations of bacteria in each sample were determined as described previously (Hsieh et al., 2010). The output/input ratio of the test strain to the virulent strain, the competitive index (CI), was interpreted as the *in vivo* survival capacity. For active immunization, five-week-old BALB/cByl mice were immunized three times by once-weekly IP injection with the indicated dose of the live *magA* single mutant or *magA wbbY* double mutant. Age-matched, unimmunized control mice (10 mice per group) were primed with an equivalent volume of saline on the same schedule. After 4 weeks, immunized and unimmunized control mice were challenged with the lethal dose 1 × 10^3^ CFU of NTUH-A4528 (O1:K2). The challenged mice were observed for 28 days for mortality and clinical signs. Survival was analyzed by Kaplan-Meier analysis with a log-rank test; a *P* value <0.05 was considered to be statistically significant. For passive protection, 100 μL of serum derived from immunized and unimmunized control mice were administered to five-week-old BALB/cByl mice by IP injection. One hour after serum injection, mice were infected IP with 1 × 10^3^ CFU of NTUH-A4528 (O1:K2) or 1 × 10^6^ CFU of *E. coli* F8188-41 per mouse. Three hour after infection, the mice were euthanized and bacterial counts from liver and spleen were determined.

### Statistical analyses

Data are presented as means ± standard deviations (SDs). Statistical significance was assessed by a two-tailed Student's *t* test using Prism 5 (Graphpad) software. Prevalence was analyzed by chi-square test using SPSS version 12.0 software. Survival was analyzed by Kaplan-Meier analysis with a log-rank test. *P* values of <0.05 were considered significant.

## Results

### Isolation of *K. pneumoniae* bacteriophage O1-1

**Figure 1:**
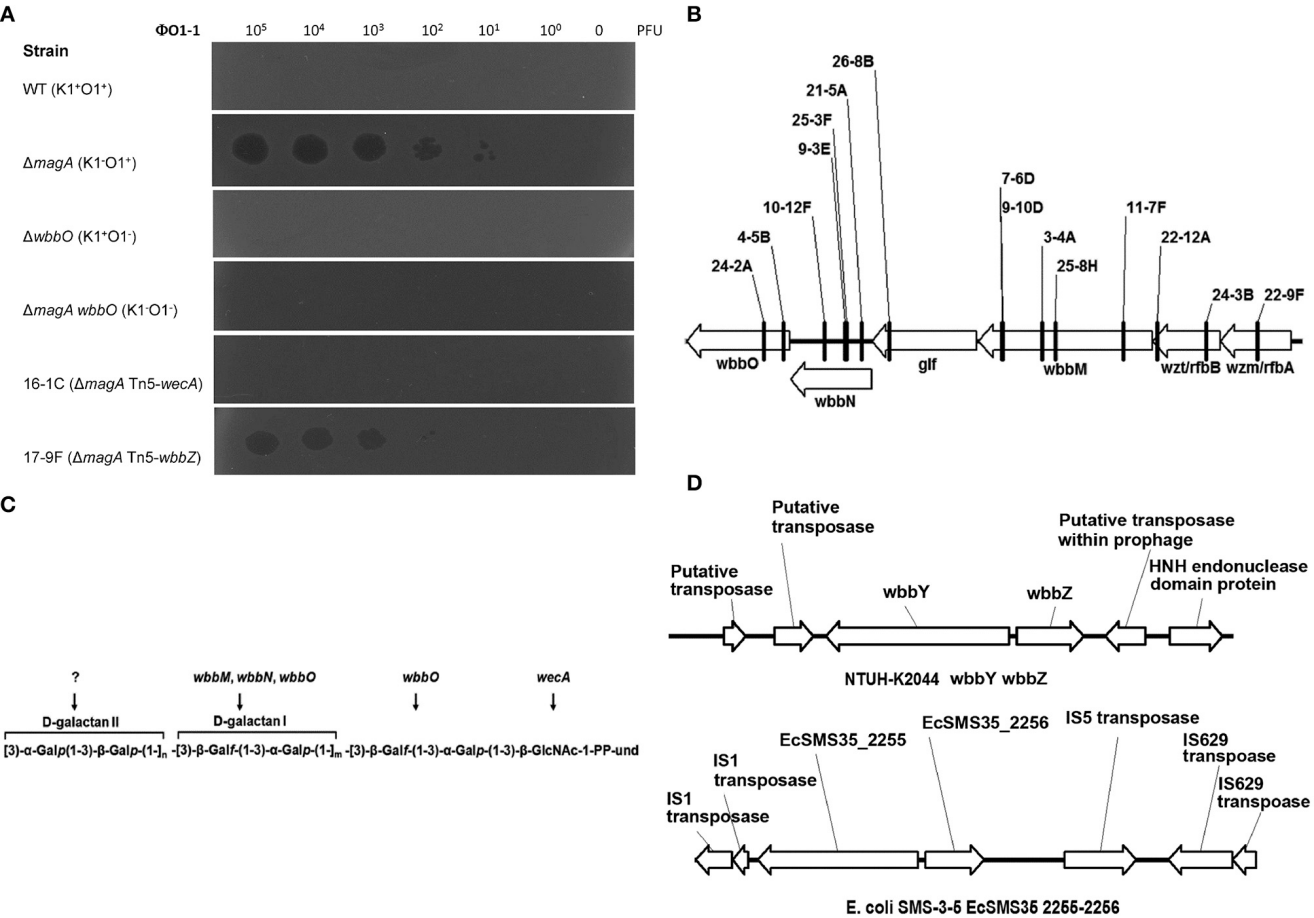
**The *K. pneumoniae* O1 mutants. (A)** The *K. pneumoniae* NTUH-K2044 wild-type, *magA* deletion, *wbbO* deletion, *magA wbbO* double-deletion mutants and *magA* deletion transposon mutant strains, 16-1C and 17-9F, were grown to mid-log phase at 37°C and spread on LB plates. Serial dilutions of SM buffer containing phage O1-1 were dotted onto the bacterial lawn and grown at 37°C for 16–18 h. PFU, plaque forming unit. **(B)** Transposon insertion sites within the *wb* cluster in the NTUH-K2044 *magA* mutants. Arrows denote the orientations of the ORFs. **(C)** The composition of *K. pneumoniae* O1 LPS and the gene responsible for each step. **(D)** Genetic organization of *wbbY, wbbZ* (or their homologs), and their flanking regions in *K. pneumoniae* NTUH-K2044 (upper) and *E. coli* SMS-3-5 (lower). Arrows denote the orientations of the ORFs.

The *magA* deletion mutant, which was derived from the NTUH-K2044 (K1:O1), lacks the outermost capsule, thereby exposing the O1 antigen (Hsieh et al., 2012). In the present study, we isolated a *K. pneumoniae* O1-1-specific bacteriophage using the *magA* mutant. A *magA* mutant lawn was successfully lysed by bacteriophage O1-1, yielding visible plaques from phage titers as low as 10 PFU (Figure 1A). In contrast, the wild-type, the *wbbO* mutant (LPS mutant), and the *magA wbbO* double-mutant (CPS- and LPS-lacking) strains were all resistant to lysis by bacteriophage O1-1, even when the viral titer was elevated to 10^5^ PFU. This result demonstrates that bacteriophage O1-1 did not infect the encapsulated *K. pneumoniae* bacteria, presumably due to masking of the receptor by CPS.

### Screening and identification of bacteriophage O1-1-resistant mutants

We constructed a *magA* deletion strain transposon library containing 2500 mutants to screen for genes associated with LPS biosynthesis. Phage O1-1-resistant mutants derived from the *magA* mutant were classified into two types, distinguished by their sensitivity to the O1-1 phage on spot tests. Among 17 phage O1-1-resistant mutants, 16 isolates (94%) were fully resistant to lysis by phage O1-1, even when the viral titer was elevated to 10^5^ PFU. In Figure 1A, this class is represented by the 16-1C mutant. In contrast, 1 of the 17 phage O1-1-resistant mutants, designated 17-9F, showed visible plaques when the viral titer was raised to 10^2^ PFU (Figure 1A). The transposon-insertion sites of these 17 phage O1-1-resistant mutants were determined by semi-random PCR and DNA sequencing. The resulting sequences were analyzed by comparison to the genome sequence of strain NTUH-K2044 (accession number AP006725). The analyses showed that 15 mutants harbored insertions within the *wb* cluster: five in *wbbM*, four in *wbbN*, two in *wbbO*, two in *wzt*, one in *wzm*, and one in *glf* (Table 1, and Figure 1B). Mutant 16-1C had an insertion in *wecA*, which initiates LPS biosynthesis by catalyzing the transfer of GlcNAc-1-phosphate onto undecaprenyl phosphate, generating undecaprenyl-pyrophosphoryl-GlcNAc (Figure 1C) (Lehrer et al., 2007). Mutant 17-9F was disrupted in a hypothetical protein-encoding gene; consistent with The Bacterial Polysaccharide Gene Nomenclature scheme (BPGN) (Reeves et al., 1996), this ORF is designated as *wbbZ* (Figure 1D).

**Table 1:** **LPS mutants in the *K. pneumoniae* Δ*magA* transposon mutant library**.

**Mutant**	**Probable interrupted gene**	**Function/probable function**	**Locus tag (NTUH-K2044 ORF)**	**Gene size (bp)**	**Tn insertion point**
3-4A	*wbbM*	Putative glycosyltransferase	KP1_3696	1908	1207
4-5B	*wbbO*	Bifunctional galactosyltransferase	KP1_3693	1143	70
7-6D	*wbbM*	putative glycosyltransferase	KP1_3696	1908	1647
9-3E	*wbbN*	Putative glycosyltransferase	KP1_3694	894	293
9-10D	*wbbM*	Putative glycosyltransferase	KP1_3696	1908	1635
10-12F	*wbbN*	Putative glycosyltransferase	KP1_3694	894	517
11-7F	*wbbM*	Putative glycosyltransferase	KP1_3696	1908	313
16-1C	*wecA*	UDP-GlcNAc:undecaprenylphosphate	KP1_0146	1104	903
		GlcNAc-1-phosphate transferase			
**17-9F**	***wbbZ***	**Hypothetical protein**	**KP1_0663**	**810**	**663**
21-5A	*wbbN*	Putative glycosyltransferase	KP1_3694	894	109
22-9F	*wzm*	Lipopolysaccharide O-antigen ABC transport system transmembrane component	KP1_3698	780	368
22-12A	*wzt*	Lipopolysaccharide O-antigen ABC transport system ATP-binding component	KP1_3697	741	689
24-2A	*wbbO*	Bifunctional galactosyltransferase	KP1_3693	1143	287
24-3B	*wzt*	Lipopolysaccharide O-antigen ABC transport system ATP-binding component	KP1_3697	741	149
25-3F	*wbbN*	Putative glycosyltransferase	KP1_3694	894	280
25-8H	*wbbM*	Putative glycosyltransferase	KP1_3696	1908	1056
26-8B	*glf*	Putative UDP-galactopyranose mutase	KP1_3695	1155	954

*The target gene or bacterial strains studied in this study are mentioned in bold*.

### Genetic analysis of *wbbY* and *wbbZ*

Analysis using the BlastX program showed that the predicted WbbZ protein shares a conserved domain with CsaB of *Bacillus subtilis*, which is a pyruvyl transferase required for polysaccharide biosynthesis (Mesnage et al., 2000). The gene adjacent to *wbbZ, wbbY*, encodes a putative glycosyltransferase that might be involved in the synthesis of oligosaccharides, polysaccharides, and glycoconjugates by transferring the sugar moiety from a sugar donor onto an acceptor (Figure 1D). The nearby flanking genes, *kp0660, kp0661*, and *kp0664*, are all putative transposase-encoding genes that are likely not associated with the biosynthesis of LPS. Moreover, WbbY and WbbZ share 99% amino acid sequence identity with EcSMS35_2255 and EcSMS35_2256 of *E. coli* SMS-3-5; these *E. coli* gene products have been conjectured to contribute to LPS biosynthesis, though the proteins were not further characterized (Fricke et al., 2008). This genetic evidence encouraged us to investigate the association between the *wbbY-wbbZ* region and LPS biosynthesis of *K. pneumoniae*.

### Glycosyltransferase wbbY is essential for the production of D-Gal II

**Figure 2:**
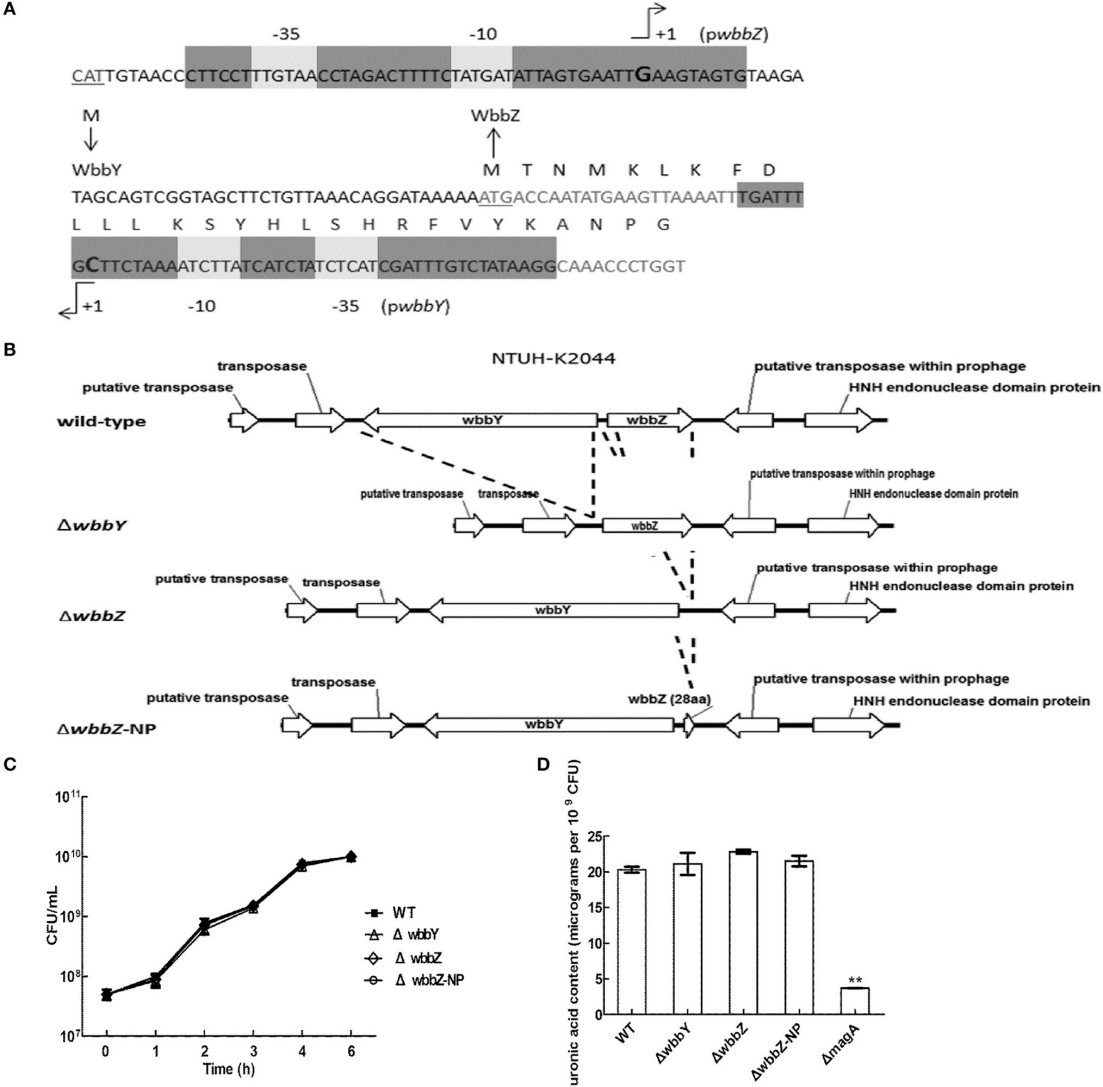
**Genetic construction, growth, and CPS production of *K. pneumoniae* NTUH-K2044 and its isogenic mutants. (A)** Mapping of transcription start sites of the *wbbY* and *wbbZ* genes. The promoter regions and the proposed −10 and −35 regions are indicated by gray shading. The transcription start sites are shown by +1 with arrowheads and in larger font. Deduced amino acid sequences (in single-letter code) are indicated beginning with an ATG start codon. **(B)** Schematic diagram representing the construction of deletion mutations in the NTUH-K2044 background. The locations and orientations of the ORFs described in this study are indicated by arrows. The dashed lines refer to joining of the regions flanking the deleted target. **(C)** Mutation of *wbbY* or *wbbZ* does not impair bacterial growth. Overnight cultures of the *K. pneumoniae* NTUH-K2044 wild-type strain, *wbbY, wbbZ*, and *wbbZ*-NP (non-polar) mutant strains were individually inoculated into fresh LB medium and grown at 37°C. The growth of bacteria was monitored periodically every hour by plating of serial dilutions on LB agar and counting CFU. The data represent the means of three independent trials; the error bars represent the standard deviations. **(D)** The amount of K1 CPS production of the *K. pneumoniae* NTUH-K2044 wild-type, and of the *wbbY, wbbZ, wbbZ*-NP, and *magA* single-mutant strains, was determined by assaying uronic acid content. The data represent the means of three independent trials; the error bars represent the standard deviations. ^**^P < 0.01 by Student's *t*-test (compared to the wild-type strain); other comparisons were not statistically significant (*P* ≥ 0.05).

The sequence features of the *wbbY-wbbZ* region suggested that *wbbY* and *wbbZ* are transcribed as a divergent gene pair; however, these two genes are only separated by a very short intergenic region consisting of 97 base-pairs between the postulated ATGs of these two ORFs. To avoid the polar effects resulting from the single mutations, that is, that deletion of *wbbY* affected the function of *wbbZ* and vice versa. Therefore, we determined the transcription initiation sites of both the *wbbY* and *wbbZ* genes by 5′-RACE (5′-Rapid Amplification of cDNA Ends) method. The transcription start site and promoter region of *wbbZ* were located within the short intergenic region between the *wbbY* and *wbbZ* genes. In contrast, the transcription start site and promoter region of the *wbbY* gene were located in the coding sequences of *wbbZ* (Figure 2A).

**Figure 3:**
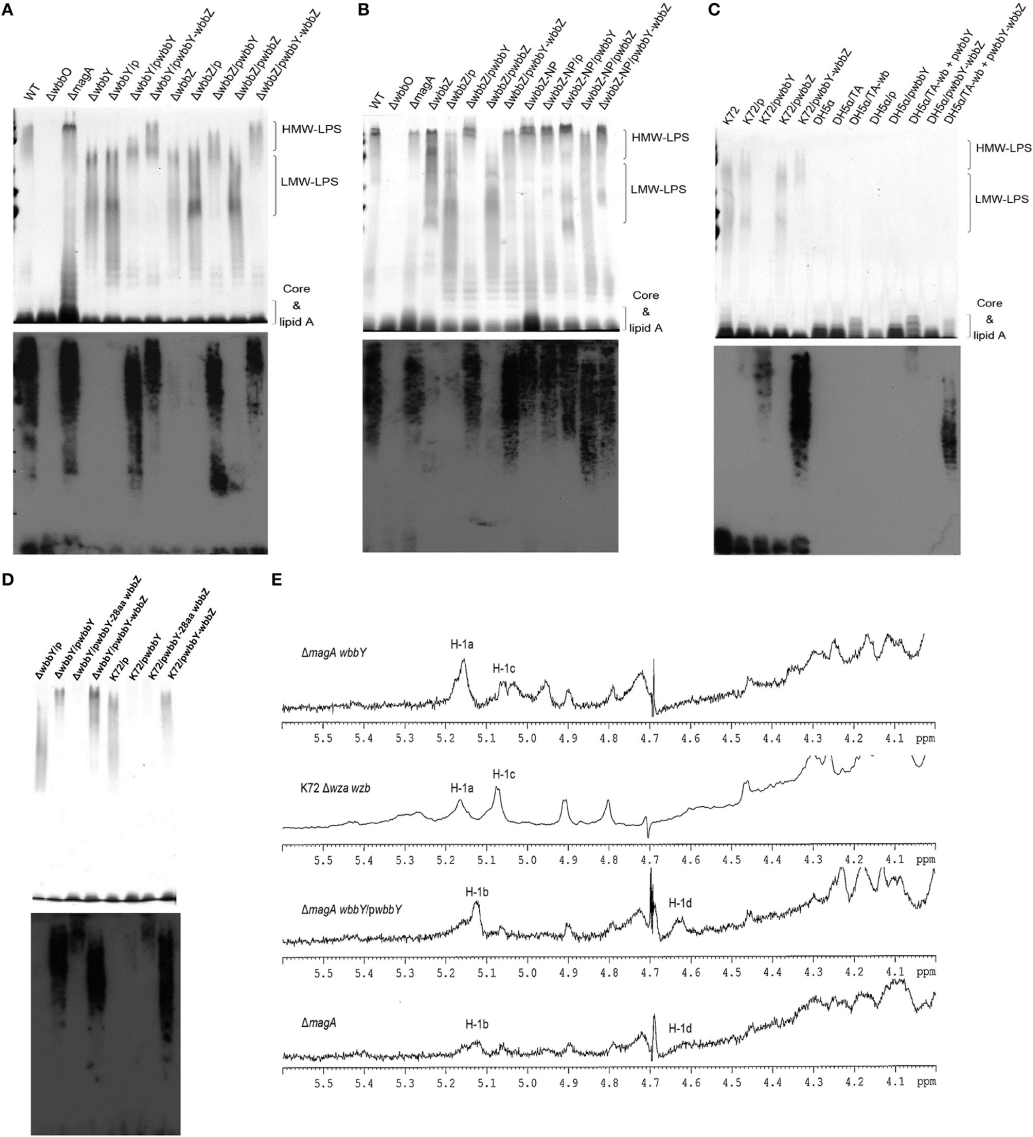
**The LPS profiles of *K. pneumoniae* NTUH-K2044 and its isogenic mutants. (A)** EPS specimens were prepared from *K. pneumoniae* NTUH-K2044, from *wbbY* and *wbbZ* single mutants, and from the same mutants harboring complementation plasmids. Extracts from normalized bacterial suspensions (10^8^ CFU) were separated by SDS-PAGE and visualized by silver staining (upper) or immunoblotting with anti-D-Gal II antiserum (lower). HMW-LPS, high-molecular-weight LPS. LMW-LPS, low-molecular-weight LPS. **(B)** EPS specimens were prepared from *K. pneumoniae* NTUH-K2044, from *wbbZ* and *wbbZ*-NP single mutants, and from mutants harboring complementation plasmids. Extracts from normalized bacterial suspensions (10^8^ CFU) were separated by SDS-PAGE and visualized by silver staining (upper) or immunoblotting with anti-D-Gal II antiserum (lower). **(C,D)** EPS extracted from *Klebsiella* K72:O2, *E. coli* DH5α, and *K. pneumoniae wbbY* mutant strains harboring the indicated plasmids were separated by SDS-PAGE and the LPS patterns were visualized by silver staining (upper) or immunoblotting with anti-D-Gal II antiserum (lower). **(E)** The ^1^H-NMR spectra of the LPS of the *wbbY* mutant. LPS specimens were prepared from the *Klebsiella* K72:O2 capsule-deficient mutant (K72Δ*wza wzb*), the *K. pneumoniae* NTUH-K2044 *magA* deletion strain, the *magA wbbY* double-deletion mutants, and the *magA wbbY* double-deletion strain containing the *wbbY*-expressing plasmid. The four D-galactosyl residues are indicated (a to d) according to decreasing anomeric ^1^H shifts.

To confirm our phage screening results, we constructed in-frame deletion mutant of *wbbY, wbbZ*, a non-polar deletion mutant of *wbbZ* that retained the upstream end of the *wbbZ* ORF (encoding 28 N-terminal amino acids of WbbZ, and corresponding to the DNA sequences of the presumed minimal *wbbY* promoter) and examined the production of CPS and LPS in these three mutants (Figure 2B). Growth rate and CPS production by in-frame deletion of *wbbY, wbbZ* or the non-polar *wbbZ* mutant strain was similar to that of the wild-type strain (Figures 2C,D). In the case of LPS expression, the wild-type and the *magA* mutant strains displayed similar patterns in both silver staining and immunoblotting, producing both HMW-LPS and LMW-LPS (Figure 3A). In contrast, the *wbbO* mutant strain completely lost its ability to produce O-antigen, displaying only the core and lipid-A in silver staining. The *wbbY* mutant was completely depleted in HMW-LPS. The in-frame *wbbZ* deletion mutant was impaired in the production of HMW-LPS, such that HMW-LPS was detected only at low levels. Unexpectedly, the non-polar *wbbZ* mutant expressed HMW-LPS and the molecule was detected by anti-D-Gal II antiserum (Figure 3B).

To compensate for the deficiency in D-Gal II synthesis, we transformed the *wbbY* and *wbbZ* single-mutant strains with plasmids containing either (i) *wbbY*, (ii) *wbbZ* with the intergenic region between these two genes, or (iii) the *wbbY*-*wbbZ* region, and then examined HMW-LPS production in the transformants. Compared to the vector control, the LPS of *wbbY* mutant harboring the *wbbY*-expressing plasmid shifted to a region of higher molecular weight, and this molecule was detected by anti-D-Gal II antiserum (Figure 3A). An even higher molecular weight LPS was obtained by introducing the *wbbY*-*wbbZ*-expressing plasmid into the *wbbY* mutant; the size of the LPS produced by this strain resembled that of the wild-type strain. Additionally, introduction of the *wbbY*-expressing plasmid resulted in a shift to a higher molecular weight of LPS in the *wbbZ* mutant. The *wbbZ*-expressing plasmid did not compensate for the lack of production of HMW-LPS in the *wbbZ* mutant. As expected, the *wbbY*-*wbbZ*-expressing plasmid fully restored the production of LPS in the *wbbZ* mutant. These results indicated that the deletion of the *wbbZ* gene have additional polar effects on *wbbY*, presumably by removing the promoter of *wbbY*. Thus, these results implied that only *wbbY* was essential for the production of D-Gal II in the *K. pneumoniae* O1 strain.

### *wbbY* and *wbbZ* are sufficient for the biosynthesis of D-Gal II

To investigate whether the *wbbY*-*wbbZ* region was sufficient for the expression of D-Gal II, we individually introduced plasmids containing (i) *wbbY*, (ii) *wbbZ* with the intergenic region between these two genes, (iii) *wbbY* with its intact promoter sequence, or (iv) the entire *wbbY*-*wbbZ* region into the *Klebsiella* K72 reference and the *wbbY* single-mutant strains. The *Klebsiella* K72 reference strain is an isolate that produces O2 antigen, which is composed only of D-Gal I and runs as LMW-LPS and is not recognized by anti-D-Gal II antiserum (Figure 3C). Introduction of a plasmid carrying *wbbY* with its intact promoter sequence enabled the K72 and the *wbbY* single-mutant strains to produce partial D-Gal II antigen of increased molecular weight that was weakly detected by anti-D-Gal II antiserum (Figure 3D). Interestingly, the introduction of the entire *wbbY*-*wbbZ* region into the K72 strain enabled the bacterium to produce D-Gal II. This results parallels the full restoration of D-Gal II antigen production to the *wbbY* mutant strain upon complementation with the plasmid containing the entire *wbbY*-*wbbZ* region.

Additionally, we compared the ^1^H-NMR spectra of LPS obtained from the *wbbY*-deletion mutant, from the *wbbY* mutant harboring the *wbbY*-expressing plasmid, and from the K72 strain. To rule out the possibility of CPS contamination during LPS analysis, we constructed these mutants in the K-antigen-deficient background. Consistent with the previous NMR assignment (Whitfield et al., 1991), we found that the LPS of the *magA wbbY* double-deletion mutant strain contained primarily D-Gal I [H-1a: -3)-β-D-Gal*f*-(1- at 5.18 ppm) and H-1c: -3)-α-D-Gal*p*-(1- at 5.06 ppm], resembling the LPS derived from the K72 *wza wzb* double-deletion mutant strain (D-Gal I). In contrast, the LPS of the *magA wbbY* double-deletion mutant harboring the *wbbY*-expressing plasmid and that of the *magA* mutant strain contained both D-Gal II [H-1b: -3]-α-D-Galp-(1- at 5.15 ppm and H-1d: -3)-β-D-Galp-(1- at 4.66 ppm] and D-Gal I (Figure 3E). In addition, we tried to express the *K. pneumoniae* O1 antigen in *E. coli*. Co-transformation of *E. coli* DH5α with two plasmids containing the *wb* cluster and the entire *wbbY*-*wbbZ* region enabled the bacterium to express a typical smooth lipopolysaccharide that could be detected by anti-D-Gal II antiserum, though the molecular weight was slightly lower than that produced in *K. pneumoniae* (Figure 3C). No HMW-LPS or smooth LPS was detected in those bacteria containing plasmids carrying the *wb* cluster only or with vector alone. Taken together, these genetic and chemical analyses indicate that both *wbbY* and *wbbZ* were sufficient for the expression of D-Gal II.

### Prevalence of the D-Gal II antigen in *K. pneumoniae* clinical isolates

In order to detect the production of D-Gal II, we generated and evaluated antiserum raised against LPS D-Gal II by immunization of a rabbit with the unencapsulated *magA* mutant (K^−^_1_ O_1_). Antiserum raised against the *magA* mutant reacted with all (20 of 20) of randomly selected *Klebsiella* reference strains, which are known to belong to the O1 serotype and produce D-Gal II. In contrast, this antiserum did not react with the *Klebsiella* K72 strain, an isolate that is known to belong to the O2 type and produce D-Gal I only (Figure 3C). Therefore, the antiserum isolated from the *magA*-mutant-immunized rabbit is specific to D-Gal II. Using this tool, we compared the distribution of D-Gal II by immunoblotting of samples from 74 clinical isolates of *K. pneumoniae*, including 42 PLA strains and 32 non-tissue-invasive strains. The D-Gal II-containing strains accounted for 63.5% of all clinical isolates, with significant differences in representation among the strains that caused PLA (38/42; 90.5%) and those that were not tissue-invasive (9/32; 28.1%) (*P* < 0.0001, chi-squared test).

### Occurrence of wbbY and wbbZ homologs in the *E. coli* O19 strain and other gram-negative bacteria

**Figure 4:**
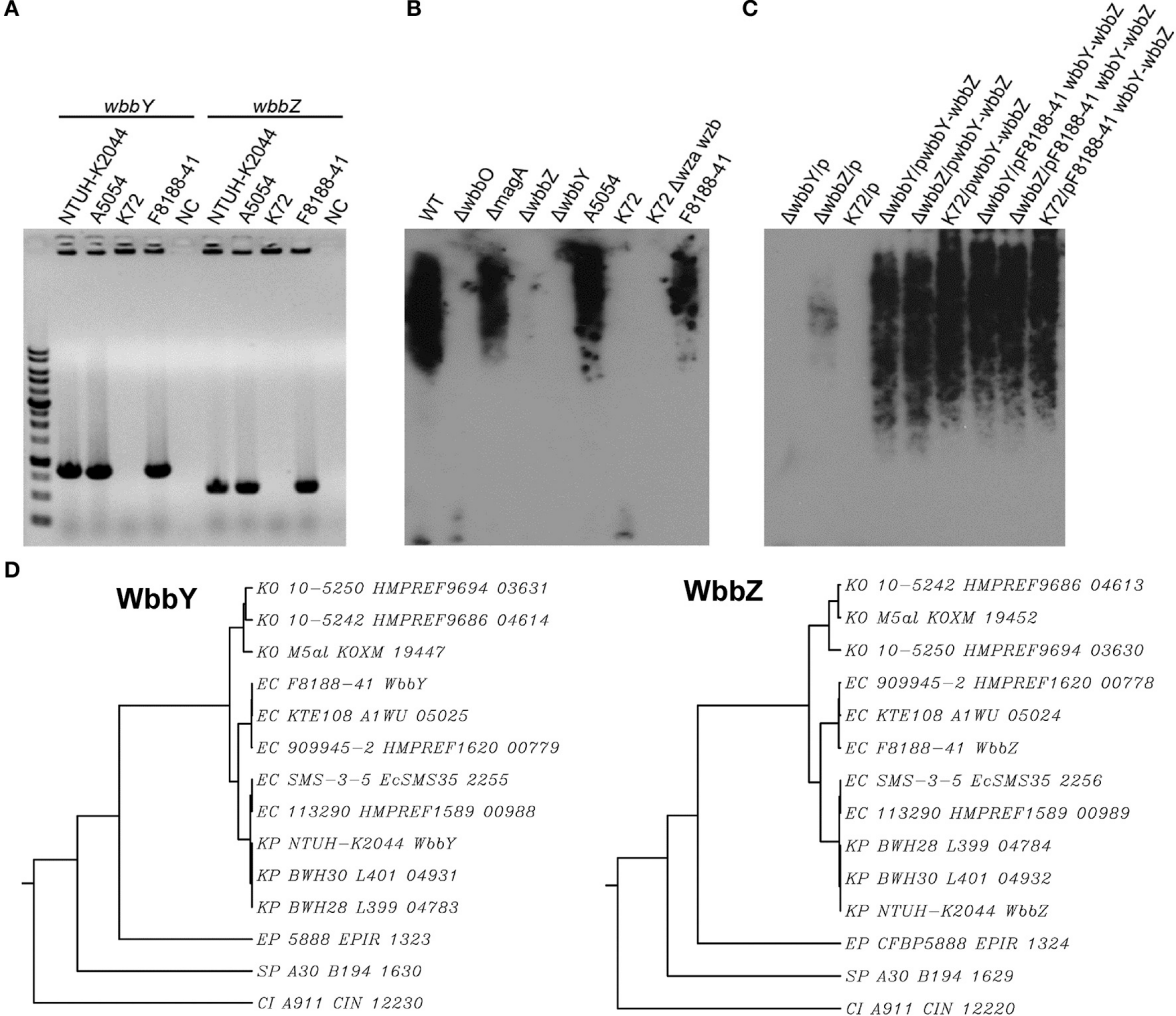
**Analysis of WbbY and WbbZ homologs in *K. pneumoniae* and other Gram-negative bacteria. (A)** PCR detection of *wbbY* (830 bp) and *wbbZ* (583 bp)—like genes. lane M, 1 kb DNA ladder; NC, no-template control. **(B)** EPS specimens were prepared from *K. pneumoniae* NTUH-K2044; isogenic mutants of K2044; strain A5054; the *Klebsiella* K72 wild type; K72 *wza wzb* double mutant; and *E. coli* O19 ab K^−^:H7 F8188-41. Extracts from normalized bacterial suspensions (10^8^ CFU) were separated by SDS-PAGE and visualized by immunoblotting with anti-D-Gal II antiserum. **(C)** EPS extracted from the *wbbY* and *wbbZ* single-mutant and *Klebsiella* K72:O2 strains harboring the indicated plasmid was separated by SDS-PAGE and the LPS patterns were analyzed by immunoblotting with anti-D-Gal II antiserum. **(D)** Phylogenetic analysis of WbbY and WbbZ homologs from different Gram-negative bacteria using the SMART (Simple Modular Architecture Research Tool) database.

A previous study indicated that the *E. coli* O19 antigen is recognized by an anti-*Klebsiella* O1 monoclonal antibody (McCallum et al., 1989; Fricke et al., 2008). We therefore investigated the link between the *wbbY* and *wbbZ* genes and structures based on D-Gal II synthesis in the *E. coli* O19-type bacterium. Specifically, we carried out PCR analysis using primer pairs located within the *wbbY* and *wbbZ* genes. Positive signals were obtained from the acapsulated *E. coli* O19ab K^−^:H7 strain, F8188-41; the amplicons were of the same sizes as those obtained from *K. pneumoniae* NTUH-K2044 and A5054, which are known O1-type strains (Figure 4A). We then carried out immunoblotting analysis using the anti-D-Gal II antiserum. All *wbbY-, wbbZ*-positive strains, including F8188-41, showed positive signals. In addition, D-Gal II was immunologically detected in all of the *wbbY* and *wbbZ* single-mutant and the K72 harboring the F8188-41 *wbbY*-*wbbZ*-expressing plasmid strains (Figures 4B,C). These results suggested that D-Gal II is present in the LPS of the *E. coli* O19 group. Moreover, we also determined the DNA sequences of the *wbbY* and *wbbZ* genes from F8188-41, which were clustered on the chromosome and showed 95% DNA sequence identity compared with those from the NTUH-K2044 and another O19-type *E. coli* strain, SMS-3-5. The WbbY and WbbZ homologs from *K. pneumoniae* NTUH-K2044 and *E. coli* SMS-3-5 showed 99% protein sequence identity and belong to the same branch by phylogenetic tree analysis (Figure 4D); the *E. coli* F8188-41 homologs showed lower protein sequence similarity and sorted as a separate phylogenetic branch. To investigate the presence of WbbY and WbbZ homologs in other bacteria, we searched the NCBI database of encoded sequences from other bacterial genomes by BLASTp. WbbY and WbbZ homologs were detected in another member of the *Klebsiella* genus (*K. oxytoca*) and in four other more distantly related Gram-negative bacteria (*E. coli, Erwinia piriflorinigrans, Serratia plymuthica*, and *Commensalibacter intestine*). In each of these species, the *wbbY* and *wbbZ* gene were adjacently located on the respective chromosome and also as divergent transcription units (Table 2). These observations suggested that the D-Gal II structure described in the present study also may be present in Gram-negative bacteria of multiple genera.

**Table 2:** **Co-existence of WbbY and WbbZ homologs in bacterial genomes**.

**Strain**	Homolog of ^*^					
	**WbbY**			**WbbZ**		
	**Locus tag**	**Length (aa)**	**Identity (%)**	**Locus tag**	**Length (aa)**	**Identity (%)**
***K. pneumoniae^†^***						
**NTUH-K2044**	**WbbY**	**740**	**100**	**WbbZ**	**269**	**100**
BWH 28	L399_04783	740	100	L399_04784	269	100
BWH 30	L401_04931	740	100	L401_04932	269	100
***K. oxytoca***						
10-5242	HMPREF9686_04614	740	93	HMPREF9686_04613	269	89
10-5250	HMPREF9694_03631	740	93	HMPREF9694_03630	269	90
M5al	KOXM_19447	648	93	KOXM_19452	269	90
***E. coli***						
**SMS-3-5**	**EcSMS35_2255**	**740**	**99**	**EcSMS35_2256**	**269**	**99**
113290	HMPREF1589_00988	740	99	HMPREF1589_00989	269	99
**F8188-41**	**WbbY**	**740**	**96**	**WbbZ**	**269**	**94**
KTE108	A1WU_05025	740	96	A1WU_05024	269	94
909945-2	HMPREF1620_00779	663	96	HMPREF1620_00778	269	94
***E. piriflorinigrans***						
CFBP 5888	EPIR_1323	758	67	EPIR_1324	270	66
***S. plymuthica***						
A30	B194_1630	736	63	B194_1629	270	63
***C. intestine***						
A911	CIN_12230	886	53	CIN_12220	266	55

* *E value < 10^−10^*.

† *100% identity of WbbY and WbbZ homologs in the NTUH-K2044 and other K. pneumoniae starins were shown. The target gene or bacterial strains studied in this study are mentioned in bold*.

### D-Gal II is required for *K. pneumoniae's* serum resistance and virulence *in vivo*

**Figure 5:**
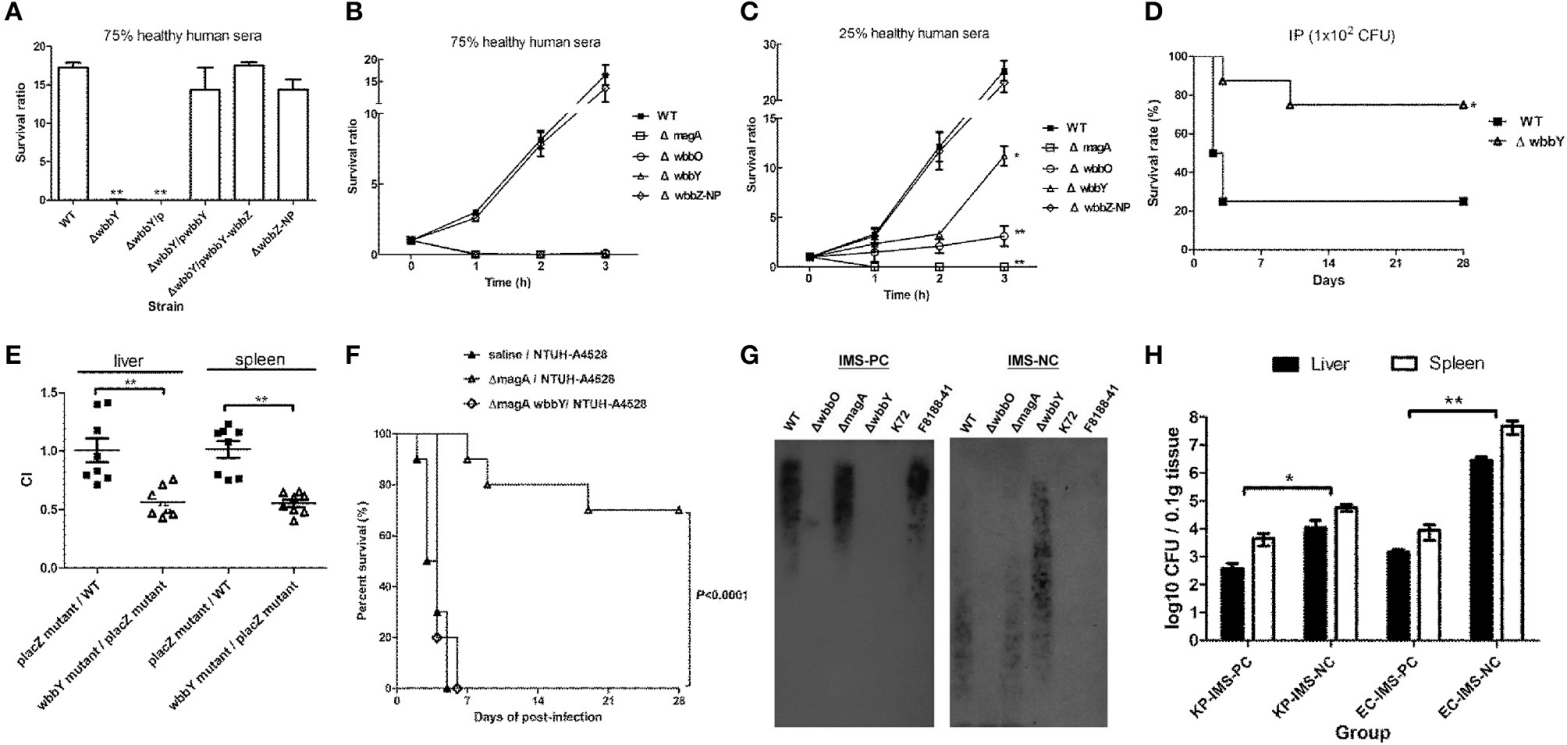
**Serum sensitivity and virulence of NTUH-K2044 and its isogenic mutants. (A–C)** Serum sensitivity assays of resistance to killing by non-immune healthy human serum of the NTUH-K2044 wild-type; *wbbY, wbbZ, wbbZ*-NP, *magA*, and *wbbO* single-deletion mutants; and the respective complemented strains. The data represent the means of three independent trials; the error bars represent the standard deviations. A mean survival ratio ≥1 corresponds to serum resistance. ^**^P < 0.01 or ^*^P < 0.05 by Student's *t* test (compared to the wild-type strain). **(D)** Eight mice per group were infected with the NTUH-K2044 wild-type and the *wbbY* mutant strains at an intraperitoneal (IP) dose of 1 × 10^2^ CFU/animal. Survival of mice was monitored for 4 weeks. ■, NTUH-K2044; △, *wbbY* mutant (*wbbY* vs. parent, *P* = 0.024; log-rank test). **(E)** Survival of the isogenic *K. pneumoniae wbbY* (open triangles) mutant in the *in vivo* bacterial competition model, using competition against the fully virulent p*lacZ* mutant in groups of eight mice. The ratio of *lacZ*^+^ or *lacZ*^−^ *K. pneumoniae* (close squares) colonies in the contents of the spleen and liver was determined from a single mouse at sacrifice (24 h post-infection). Each symbol represents the competitive index (CI) for each inoculum; and the medians and SDs of the values are shown (for the wild-type strain group vs. p*lacZ* mutant group, the CI of spleen and liver were 1.016 ± 0.206 and 1.009 ± 0.286, respectively; *wbbY* mutant group vs. p*lacZ* mutant group, the CI of spleen and liver were 0.553 ± 0.089 and 0.564 ± 0.123, respectively; *P* = 0.001, Wilcoxon's signed rank test). **(F)** Survival of mice immunized with *magA* mutant and then challenged with NTUH-A4528 (O1:K2). Mice (10 per group) were inoculated three times by once-weekly IP injections with 1 × 10^6^ CFU of *magA* mutant or *magA wbbY* double-mutant strain. Age-matched, unimmunized control mice were inoculated with saline. On the fourth week, immunized or unimmunized groups were challenged with NTUH-A4528 (1 × 10^3^ CFU per animal, IP). Survival was assessed for 28 days following infection. ▲, non-immunized, NTUH-A4528 challenged; △, *magA* mutant immunized, NTUH-A4528 challenged; ◊, *magA wbbY* double mutant immunized, NTUH-A4528 challenged. By log-rank test: *P* < 0.0001, *magA/*NTUH-A4528 vs. non-immune/NTUH-A4528 or *magA wbbY*/NTUH-A4528. **(G)** Immune response of anti-D-Gal II IgG in *magA* mutant-immunized mice. Immunoblots developed with *magA* mutant immune mouse serum (1:500) or *magA wbbY* double-mutant immune mouse serum (1:500). **(H)** Anti-D-Gal II specific antibodies reduce growth of NTUH-A4528 (O1:K2) *K. pneumoniae* and *E. coli* F8188-41 (O19) in mice. Groups of 4 BALB/c mice were treated with *magA* mutant immune mouse serum (IMS–positive control, IMS-PC) or with *magA wbbY* double-mutant immune mouse serum (IMS–negative control, IMS-NC). One hour after injection, mice were infected with 1 × 10^3^ CFU of the NTUH-A4528 (KP-IMS-PC and KP-IMS-NC) or 1 × 10^6^ CFU of *E. coli* F8188-41 (EC-IMS-PC and EC-IMS-NC) by IP injection. Three hours after infection, the numbers of bacteria in the liver and spleen were determined. Log_10_ CFU was standardized per 0.1 gram wet organ weight. The black and white bars represent the means for each group for liver and spleen, respectively. Data are presented as means ± *SD*. ^**^P < 0.01 or ^*^P < 0.05 by Student's *t* test (KP-IMS-PC vs. KP-IMS-NC and EC-IMS-PC vs. EC-IMS-NC).

Serum killing assays were performed on the *K. pneumoniae wbbY* and non-polar *wbbZ* single-deletion mutants to determine the mutants' sensitivities to serum bactericidality. Exposure to human serum (75% human serum for 3 h) resulted in higher levels of killing in the *wbbY* mutant than in the wild-type and non-polar *wbbZ* mutant strains (Figure 5A); the wild-type and non-polar *wbbZ* mutant strains exhibited similar levels of resistance to serum killing. Moreover, introduction of plasmid carrying either *wbbY* alone or the entire *wbbY*-*wbbZ* region successfully restored serum resistance levels to the *wbbY* mutant strain. This result confirmed that resistance to serum killing correlated with the production of D-Gal II.

To delineate the contribution of CPS, D-Gal I, and D-Gal II to *K. pneumoniae*'s serum resistance, the survival ratio of the wild-type, *magA, wbbO, wbbY* and non-polar *wbbZ* single-deletion mutant strains after killing by 75 and 25% human serum was assessed at one-hour intervals through 3 h of exposure (Figures 5B,C). Mutation of *magA* dramatically reduced the serum resistance of the NTUH-K2044, presumably because the mutant lacked K1 antigen. As expected, w*bbO* mutation resulted in the loss of O1 antigen; the mutant exhibited elevated susceptibility to serum killing compared to the wild-type, *wbbY*, and non-polar *wbbZ* mutant strains. The *wbbY* mutant, which lacked D-Gal II, showed more susceptible to serum killing than the wild-type and non-polar *wbbZ* mutant strains. These results demonstrated that the sensitivity to serum killing of the K1:O1 *K. pneumoniae* strain depended on the integrity of bacterial surface polysaccharides.

To evaluate the contribution of the LPS D-Gal II of *K. pneumoniae* in bacteremia, we challenged mice with the same dose (1 × 10^2^ CFU) of the wild-type and *wbbY* deletion-mutant strains. Upon IP infection of mice, the *wbbY* mutant showed significantly reduced virulence compared to the parent strain (*P* = 0.024, Figure 5D). Previously, we generated an isogenic *lacZ* mutant with its promoter deletion and used it as the wild-type strain in the competition assay (Hsieh et al., 2010). In those experiments, no competitive advantage was observed for either the wild type or p*lacZ* mutant in the spleen and liver of BALB/c mice. Using the same assay in the present study, the *wbbY* mutant strain showed lower competitive indices than the wild type or p*lacZ* mutant (*P* < 0.001, Figure 5E). These results indicated that D-Gal II was required for *K. pneumoniae*'s serum resistance and contributed to *in vivo* virulence.

### Protective effect of anti-D-Gal II antibodies during *K. pneumoniae* infection

To evaluate the protective efficacy of immunization against D-Gal II, mice were pretreated by IP injection with either *magA* or *magA wbbY* (1 × 10^6^ CFU per dose), administered once weekly for 3 weeks. On the fourth week, the animals (in groups of 10) were challenged IP with a lethal dose of highly virulent *K. pneumoniae* NTUH-A4528 (O1:K2). Within 6 days of NTUH-A4528 infection, 100% of the unimmunized control mice or the mice pre-immunized with the *magA wbbY* double-mutant strain died. Meanwhile, 70% of the mice pre-immunized with the *magA*-mutant (K^−^_1_ O_1_) survived without any symptoms of disease through 28 days after challenge (*P* < 0.0001, Figure 5F). Mice immunized with the *magA* mutant (K^−^_1_ O_1_) showed anti-D-Gal II immunoglobulin G (IgG) production by immunoblot; mice immunized with the *magA wbbY* double mutant or unimmunized control mice did not (Figure 5G).

The results of the immunization study were consistent with a separate test in which the passive protective efficacy of anti-D-Gal II antiserum was tested. In the passive immunization study, mice were pretreated with immune mouse sera (negative and positive controls, respectively). The *in vivo* protective capacity of the sera was tested using a *K. pneumoniae* or an *E. coli* septicemia infection model, whereby the mice were inoculated IP with 1 × 10^3^ CFU of the encapsulated *K. pneumoniae* NTUH-A4528 (O1:K2) or 1 × 10^6^ CFU of the acapsulated *E. coli* F8188-41 (O19) strain. The bacterial load was determined in both the liver and spleen at 3 h post-infection. Compared to animals pre-treated with the *magA wbbY* double-mutant immune mouse serum (IMS-NC), mice pretreated with the *magA* mutant immune mouse serum (IMS-PC) had significantly reduced bacterial loads (Figure 5H). These results indicated that in both active and passive immunization studies, D-Gal II-specific antiserum was able to reduce bacterial dissemination and protect against infection by encapsulated O1:K2 PLA-associated *K. pneumoniae* and by D-Gal II-containing *E. coli* in mouse models of septicemia.

## Discussion

In this study, we identified 17 *K. pneumoniae magA*-deletion transposon mutants resistant to the bacteriophage O1-1. Among these mutants, 16 mutants were disrupted in well-characterized LPS-biosynthesis genes: 15 were in the *wb*-cluster genes, and 1 was in *wecA*, indicating that a bacteriophage screening method was very useful. The last mutant represented a transposon insertion into *wbbZ* (formerly designated *kp0663*), a locus located well away from the *wb* cluster [located in a region formerly designated *kp3698* (*wzm*) to *kp3693* (*wbbO*)]. This result was consistent with the previous finding that genes involved in the expression of D-Gal II were not closely linked to the *wb* cluster (Whitfield et al., 1991).

The gene adjacent to *wbbZ, wbbY*, has apparent carbohydrate transferase functions and conserved among D-Gal II synthesizing organisms. The sequence features of this region suggested that these two genes are adjacently located on the respective chromosome and also as divergent transcription units; however, they are only separated by a very short intergenic region. Indeed, mapping of the transcription initiation sites of the two genes of the NTUH-K2044 revealed that the transcription start site of the *wbbY* gene was located within the coding region of *wbbZ*, apparent overlap of regulatory sequences so that *wbbZ* insertion has polar effects on *wbbY*. More detailed studies will be needed to clarify the regulation and interaction of these overlapping promoters.

Silver staining revealed that the *wbbY* mutant had enriched LMW-LPS, but lacked HMW-LPS. *Trans*-complementation of the *wbbY* mutation by the *wbbY*-expressing plasmid resulted in the production of LPS with a molecular weight between those of the LPS of the wild-type and mutant strains; this intermediate molecular weight product was detected by anti-D-Gal II antiserum. These findings suggest that *wbbY* encodes a glycosyltransferase that has a central role in the elongation of D-Gal II. The D-Gal II structure is the difference between the LPS composition of *K. pneumoniae* serotypes O1 and O2. Expression of the entire *wbbY*-*wbbZ* region in a *Klebsiella* O2 strain enabled the bacterium to express HMW-LPS that was recognized by anti-D-Gal II antiserum. Furthermore, co-transformation of *E. coli* DH5α with two plasmids carrying the *K. pneumoniae wb* cluster and the *wbbY*-*wbbZ* regions permitted the *E. coli* K-12 recipient (which normally produces rough LPS consisting of only lipid A-core) to produce a typical smooth LPS ladder. These pieces of evidence support the contention that both the *wbbY* and *wbbZ* genes are sufficient for the biosynthesis of D-Gal II. Determination of their precise functions in D-Gal II assembly awaits further study. The current data suggests that WbbZ participates in some other way, perhaps involving termination of the elongation process, following the step catalyzed by WbbY.

Sequence analysis also showed that WbbY and WbbZ shared a high degree of sequence similarity with two putative LPS biosynthesis genes of SMS-3-5, an *E. coli* O19:H34 strain (McCallum et al., 1989; Fricke et al., 2008). Interestingly, an early study indicated that the *E. coli* O19 antigen could be recognized by a *Klebsiella* O1 antigen-specific monoclonal antibody (McCallum et al., 1989; Fricke et al., 2008). This connection led us to the reasonable assumption that the *E. coli* O19 group has an O-antigen structure similar to that of *Klebsiella* O1, as well as the genetic apparatus required for synthesis of O antigen. That is exactly what we demonstrate in this study: a genetic apparatus for D-Gal II biosynthesis is present in the *E. coli* O19 group. Therefore, it is conceivable that WbbY and WbbZ participate in the assembly of D-Gal II-containing LPS in the *K. pneumoniae* serotype O1 and the *E. coli* serotype O19 strains.

Previous studies reported that D-Gal I and structures based on D-Gal I are found in a variety of *Klebsiella* O serotypes, as well as in *Serratia plymuthica, S. marcescens* O16 and O20, and *Pasteurella hemolytica* serotypes 4 and T10 (Oxley and Wilkinson, 1989; Richards and Leitch, 1989; Whitfield et al., 1992; Aucken et al., 1993; Kelly et al., 1993). However, D-Gal II is only found in O-antigen from *Klebsiella* serotypes O1 and O8, and in *S. plymuthica* (Aucken et al., 1993; Kelly et al., 1993). In fact, database searches performed as part of the present study indicate WbbY and WbbZ homologs are present in several bacterial genera, especially within the *Enterobacteriaceae*. Taken together, it is conceivable that WbbY and WbbZ homologs participate in the assembly of D-Gal II-containing LPS in a wider and previously unappreciated range of Gram-negative bacteria.

Deletion of the *wbbY* gene impaired the survival of the NTUH-K2044 in human serum, presumably because the mutant lacked D-Gal II. This result is consistent with an earlier finding that the presence of D-Gal II is required for the resistance of *K. pneumoniae* to serum killing (McCallum et al., 1989). The importance of the bacterial surface EPS to serum resistance of the O1:K1 *K. pneumoniae* strain is ranked in decreasing order from K1 antigen, O1 antigen, D-Gal I, and D-Gal II. Upon *in vivo* infection, the D-Gal II-deficient *K. pneumoniae* mutant is less virulent than the parental strain. Loss of D-Gal II production in *K. pneumoniae* restricts the spread of the bacteria to distant organs, reducing mice mortality. Thus, these findings indicate that mutation of the *K. pneumoniae* gene coding for D-Gal II biosynthetic machinery results in changes in serum resistance; reducing bacterial dissemination and colonization into deep organs.

The O antigen is the most external component of the LPS. In the present study, D-Gal II-specific antiserum was obtained from a rabbit immunized with a *magA* mutant (K^−^_1_ O_1_) strain. This result indicates that D-Gal II is the immunodominant antigen of the LPS O1 antigen of *K. pneumoniae*. The high prevalence (90.5%) of D-Gal II in PLA strains indicates that the D-Gal II-producing strains are predominant among *K. pneumoniae* isolates causing PLA. Earlier results showed that K antigen and LPS are exposed together at the cell surface in *K. pneumoniae* O1 strains harboring CPS serotype K2 (Merino et al., 1992). Clements *et al*. used *K. pneumoniae* B5055 (O1:K2), a pneumonia strain, to show that immunization with purified LPS can protect mice against lethal challenge with either O1:K2 or O2:K1 *K. pneumoniae* infection (Clements et al., 2008). In the present study, mice immunized with the *magA* mutant (K^−^_1_ O_1_) induced the production of antiserum against D-Gal II, and this antiserum had a significant protective efficacy against the PLA-associated O1:K2 strain. In passive immunization studies, D-Gal II-specific antiserum was able to limit the growth and bacterial dissemination of encapsulated O1:K2 PLA-associated *K. pneumoniae* or acapsulated O19-type *E. coli* in mouse models of septicemia. Therefore, LPS D-Gal II antigen could be a useful vaccine target conferring broad cross-protection against D-Gal II-containing Gram-negative bacterial pathogens.

In summary, bacteriophage is a powerful screening tool for identification of bacterial surface components. The gene encoding glycosyltransferase WbbY is essential for the production of D-Gal II in *K. pneumoniae*. Both WbbY and WbbZ homologs are sufficient for the biosynthesis of D-Gal II. LPS D-Gal II is more prevalent in PLA strains than in non-tissue-invasive strains. Besides the *Klebsiella* O1 strain, the *E. coli* O19 group and several other species of Gram-negative bacteria also harbor WbbY homologs, WbbZ homologs, and LPS structures based on D-Gal II. Mutation of the gene coding for biosynthesis of the D-Gal II antigen in *K. pneumoniae* changes serum resistance; reduces bacterial dissemination and colonization into deep organs; and decreases mouse mortality upon infection with *K. pneumoniae*. D-Gal II is the immunodominant antigen of LPS and immunization of mice against D-Gal II provides protection against infection with an O1:K2 PLA-associated *K. pneumoniae* strain.

### Conflict of interest statement

The authors declare that the research was conducted in the absence of any commercial or financial relationships that could be construed as a potential conflict of interest.

## Abbreviations

LPS: lipopolysaccharide
D-Gal I: D-galactan I
D-Gal II: D-galactan II
EPS: exopolysaccharide
CPS: capsular polysaccharide
PLA: community-acquired pyogenic liver abscess
LMW: low-molecular-weight
HMW: high-molecular-weight
5′-RACE: 5′-Rapid Amplification of cDNA Ends
IP: intraperitoneal
PFU: plaque-forming units
CFU: colony-forming units
CI: competitive index.
