# Supplementary material for: D-galactan II is an immunodominant antigen in O1 lipopolysaccharide and affects virulence in Klebsiella pneumoniae: implication in vaccine design
Source: Front Microbiol. 2014 Nov 19;5:608. doi: 10.3389/fmicb.2014.00608 (PMC4237132; doi:10.3389/fmicb.2014.00608)
Supplement: Supplementary file 1 [file Table1.PDF]

Table S1. Bacterial strains, plasmids and primers used in this study

Bacterial strain, plasmid or primer	Genotype, sequence and / or relevant description	Reference or source
**Bacteria**		
Klebsiella pneumoniae strains		
PLA strain (42 strains)	Clinical isolate *K. pneumoniae* strains causing PLA and collected from NTUH during 1997-2003.	(Hsieh et al., 2008;Hsieh et al., 2010)
Non–tissue-invasive strain (32 strains)	Clinical isolate *K. pneumoniae* strains causing pancreatitis, biliary tract stones with cholangitis or gall bladder empyema and collected from NTUH during 1997-2003.	(Hsieh et al., 2008;Hsieh et al., 2010)
WT	NTUH-K2044, O1:K1, S^r, clinical isolate PLA strain, the parent strain for generate isogenic mutants	(Fang et al., 2004)
Δ*magA*	NTUH-K2044 isogenic mutant with deletion of *magA* gene	(Hsieh et al., 2012)
Δ*wbbO*	NTUH-K2044 isogenic mutant with deletion of *wbbO* gene	(Hsieh et al., 2012)
Δ*magA wbbO*	NTUH-K2044 isogenic mutant with double deletion of *magA* and *wbbO* genes	(Hsieh et al., 2012)
16-1C	Δ*magA* mutant containing a Tn*5*-insertion in *wecA* gene	This study
17-9F	Δ*magA* mutant containing a Tn*5*-insertion in *wbbZ* gene	This study
Δ*wbbY*	NTUH-K2044 isogenic mutant with deletion of *wbbY* gene	This study
Δ*wbbY*/p	Δ*wbbY* mutant carrying pACYC184 plasmid	This study
Δ*wbbY/*p*wbbY*	Δ*wbbY* mutant carrying pACYC184 derivative containing *wbbY* from NTUH-K2044	This study
Δ*wbbY*/p*wbbY-wbbZ*	Δ*wbbY* mutant carrying pACYC184 derivative containing *wbbY* and *wbbZ* from NTUH-K2044	This study
Δ*wbbY*/p*wbbY-28aa wbbZ*	Δ*wbbY* mutant carrying pACYC184 derivative containing *wbbY* and the upstream end of the *wbbZ* ORF (encoding 28 N-terminal amino acids of WbbZ, and corresponding to the DNA sequences of the presumed minimal *wbbY* promoter) from NTUH-K2044	This study

Δ*wbbY*/pF8188-41 *wbbY-wbbZ*	Δ*wbbY* mutant carrying pACYC184 derivative containing *wbbY* and *wbbZ* from F8188-41	This study
Δ*wbbZ*	NTUH-K2044 isogenic mutant with deletion of *wbbZ* gene	This study
Δ*wbbZ*/p	Δ*wbbZ* mutant carrying pACYC184 plasmid	This study
Δ*wbbZ/pwbbZ*	Δ*wbbZ* mutant carrying pACYC184 derivative containing *wbbZ* from NTUH-K2044	This study
Δ*wbbZ/pwbbY*	Δ*wbbZ* mutant carrying pACYC184 derivative containing *wbbY* from NTUH-K2044	This study
Δ*wbbZ/pwbbY-wbbZ*	Δ*wbbZ* mutant carrying pACYC184 derivative containing *wbbY* and *wbbZ* from NTUH-K2044	This study
Δ*wbbZ*/pF8188-41 *wbbY-wbbZ*	Δ*wbbZ* mutant carrying pACYC184 derivative containing *wbbY* and *wbbZ* from F8188-41	This study
Δ*wbbZ*-NP	NTUH-K2044 isogenic mutant with deletion of partial *wbbZ* gene, retaining 28 amino acids in N-terminal of WbbZ	This study
Δ*wbbZ*-NP/p	Δ*wbbZ*-NP mutant carrying pACYC184 plasmid	This study
Δ*wbbZ*-NP*/pwbbZ*	Δ*wbbZ*-NP mutant carrying pACYC184 derivative containing *wbbZ* from NTUH-K2044	This study
Δ*wbbZ*-NP*/pwbbY*	Δ*wbbZ*-NP mutant carrying pACYC184 derivative containing *wbbY* from NTUH-K2044	This study
Δ*wbbZ*-NP*/pwbbY-wbbZ*	Δ*wbbZ*-NP mutant carrying pACYC184 derivative containing *wbbY* and *wbbZ* from NTUH-K2044	This study
K72	*Klebsiella* O2:K72 strain 1205	Statens Serum Institute
K72 Δ*wza wzb*	K72 isogenic mutant with deletion of *wza* and *wzb* genes	This study
K72/p	K72 carrying pACYC184 plasmid	This study
K72/p*wbbY-wbbZ*	K72 carrying pACYC184 derivative containing *wbbY* and *wbbZ* from NTUH-K2044	This study
K72/p*wbbY-28aa wbbZ*	K72 carrying pACYC184 derivative containing *wbbY* and the upstream end of the *wbbZ* ORF (encoding 28 N-terminal amino acids of WbbZ, and corresponding to the DNA sequences of the presumed minimal *wbbY* promoter) from NTUH-K2044	This study
K72/pF8188-41 *wbbY-wbbZ*	K72 carrying pACYC184 derivative containing *wbbY* and *wbbZ* from F8188-41	This study
A5054	*K. pneumoniae* O1:K1 strain A5054	Statens Serum Institute

NTUH-A4528	O1:K2, S^r, clinical isolate PLA strain	(Fang et al., 2004)
Escherichia coli strains		
DH10B	F$^-$ *mcrA* Δ*(mrr-hsdRMS-mcrBC)* Φ*80* δλαχZ ΔM15 Δ*lacX74 endA1 recA1 deoR (ara leu)7697 ara*Δ*139 galU galK nupG rpsL* λ$^-$	Invitrogen
S17–1λpir	Tp^r Sm^r *recA, thi, pro, hsdR-M+RP4*: 2-Tc:Mu: Km Tn7 *λpir*	(Simon et al., 1983)
DH5α	F– Φ80*lac*ZΔM15 Δ(*lac*ZYA-*arg*F) U169 *rec*A1 *end*A1 *hsd*R17 (rK–, mK+) *pho*A *sup*E44 λ– *thi*-1 *gyr*A96 *rel*A1	Invitrogen
F8188-41	*Escherichia coli* O19ab:K-:H7 strain	Statens Serum Institute
**Plasmids**		
pGEM-T Easy	TA cloning vector; Ap^r	Promega
pKO3-Km	pKO3 derivative containing a Km resistance cassette from pUC4K into AccI site	(Hsieh et al., 2008)
TA	PCR II -TOPO cloning vector; Ap^r Km^r	Invitrogen
TA-*wb*	PCR II -TOPO derivative containing the *wb* cluster from NTUH-K2044	(Hsieh et al., 2012)
p	pACYC184 low-copy-number cloning vector; Cm^r, Tc^r	Fermentas
p*wbbY*	pACYC184 derivative containing *wbbY* gene from NTUH-K2044; Cm^r	This study
p*wbbZ*	pACYC184 derivative containing *wbbZ* gene from NTUH-K2044; Cm^r	This study
p*wbbY-wbbZ*	pACYC184 derivative containing *wbbY* and *wbbZ* genes from NTUH-K2044; Cm^r	This study
p*wbbY-28aa wbbZ*	pACYC184 derivative containing *wbbY* and the upstream end of the *wbbZ* ORF (encoding 28 N-terminal amino acids of WbbZ, and corresponding to the DNA sequences of the presumed minimal *wbbY* promoter) from NTUH-K2044; Cm^r	
pF8188-41 *wbbY-wbbZ*	pACYC184 derivative containing *wbbY* and *wbbZ* genes from F8188-41; Cm^r	This study

Primers

P1	GCGCGCTGCGCAGGGCTTTATTGATTCCATTTTTAC	Semi-Random PCR	(Chun et al., 1997)
P2	GGCCACGCGTCGACTAGTACNNNNNNNNNNGATAT	Semi-Random PCR	(Chun et al., 1997)
P3	GTACCGAGCTCGAATTCGGC	Semi-Random PCR	(Chun et al., 1997)
P4	GGCCACGCGTCGACTAGTAC	Semi-Random PCR	(Chun et al., 1997)

Km4647F	CCTGCAGGCATGCAAGCTTC	Semi-Random PCR	This study
Km2921R	GTACCGAGCTCGAATTCGGC	Semi-Random PCR	(Wu et al., 2011)
KP0662-793F	CCGCGTATTCATATTCAGC	*wbbY* deletion mutant construct	This study
KP0663-R	TCAACTTGCCGTAATAAAGC	*wbbY* deletion mutant construct<br>*wbbZ* complementation construct<br>the *wbbY*-*wbbZ* region complementation construct<br>5’RACE of *wbbZ*<br>PCR of *wbbY*-*wbbZ* genes from F8188-41<br>PCR of *wbbZ*	This study
KP0662-IF	TGTAACCCTTCCTTTGTAAC	*wbbY* deletion mutant construct<br>*wbbZ* complementation construct	This study
KP0662-IR	AGTAATATTCGGGGCCCG	*wbbY* deletion mutant construct	This study
KP0662-F	CCATCATCAACCAAGATGACC	*wbbZ* deletion mutant construct<br>*wbbZ*-NP deletion mutant construct<br>5’RACE of *wbbY* (nested PCR)	This study
KP0663+744R	AACAGCACCCGCTGGAAAAG	*wbbZ* deletion mutant construct<br>*wbbZ*-NP deletion mutant construct	This study
KP0662-P-FL	TTTTTATCCTGTTTAACAGAAGCTACC	*wbbZ* deletion mutant construct<br>*wbbY* complementation construct	This study
KP0663-IF	AAAAGGCAGCGTATAATAATAC	*wbbZ* deletion mutant construct	This study
kp0663-IFS	TGAAAAAGGCAGCGTATAATAATACGC	*wbbZ*-NP deletion mutant construct	This study
kp0663-28aN	ACCAGGGTTTGCCTTATAGAC	*wbbZ*-NP deletion mutant construct	This study
KP0662-RR’	TTATTTTAACATTGATTTCACTT	*wbbY* complementation construct<br>the *wbbY*-*wbbZ* region complementation construct<br>the *wbbY*-28aa *wbbZ* region complementation construct<br>PCR of *wbbY*-*wbbZ* genes from F8188-41	This study
kp0663NP	GCAGCCTGTCGGTATTGACAAC	5’RACE of *wbbZ* (nested PCR)	This study

kp0662-210a	TCTCGTCACAAACCCTTGAAATGG	5’RACE of *wbbY*	This study
kp0663-28aNR	TCAACCAGGGTTTGCCTTATAGAC	the *wbbY*-28aa *wbbZ* region complementation construct	This study
wza-CF	GTGAAGTTCTGGAACCAGTGG	K72 *wza wzb* deletion mutant construct	This study
wzc-R1	TCCATCATTGCAAAATGCAAACT	K72 *wza wzb* deletion mutant construct	This study
K72 wza-IR	AATGTCACATCATCAGTAA	K72 *wza wzb* deletion mutant construct	This study
K72 wza-IF	GTAACTGACCAAGGAAAAGATATAG	K72 *wza wzb* deletion mutant construct	This study
KP0662-F1	GGCTTAGCAACATTATCATCGTC	PCR of *wbbY*	This study
KP0662-RR	CTTGAAACCCGTAAAGGACT	PCR of *wbbY*	This study
KP0663+228F	AGGATTGTATTCTGAAGGTC	PCR of *wbbZ*	This study

NOTE. NTUH, National Taiwan University Hospital; PLA, community-acquired pyogenic liver abscess; S^r, serum resistance; Tp, trimethoprim; Sm, streptomycin; Km, kanamycin; Ap, ampicillin; Cm, chloramphenicol; Tc, tetracycline
